# Identifying and selecting implementation theories, models and frameworks: a qualitative study to inform the development of a decision support tool

**DOI:** 10.1186/s12911-020-01128-8

**Published:** 2020-05-14

**Authors:** Lisa Strifler, Jan M. Barnsley, Michael Hillmer, Sharon E. Straus

**Affiliations:** 1 Institute of Health Policy Management & Evaluation, University of Toronto, 155 College Street, Toronto, Ontario M5T 3M6 Canada; 2 Knowledge Translation Program, Li Ka Shing Knowledge Institute, St. Michael’s Hospital, Unity Health Toronto, 209 Victoria Street, Toronto, Ontario M5B 1W8 Canada; 3 Ontario Ministry of Health and Long-Term Care, 900 Bay Street, Toronto, Ontario M7A 1R3 Canada; 4 Department of Geriatric Medicine, University of Toronto, 27 King’s College Circle, Toronto, Ontario M5S 1A1 Canada

**Keywords:** Implementation, Theory, Model, Framework, Interviews, Decision support

## Abstract

**Background:**

Implementation theories, models and frameworks offer guidance when implementing and sustaining healthcare evidence-based interventions. However, selection can be challenging given the myriad of potential options. We propose to inform a decision support tool to facilitate the appropriate selection of an implementation theory, model or framework in practice. To inform tool development, this study aimed to explore barriers and facilitators to identifying and selecting implementation theories, models and frameworks in research and practice, as well as end-user preferences for features and functions of the proposed tool.

**Methods:**

We used an interpretive descriptive approach to conduct semi-structured interviews with implementation researchers and practitioners in Canada, the United States and Australia. Audio recordings were transcribed verbatim. Data were inductively coded by a single investigator with a subset of 20% coded independently by a second investigator and analyzed using thematic analysis.

**Results:**

Twenty-four individuals participated in the study. Categories of barriers/facilitators, to inform tool development, included characteristics of the individual or team conducting implementation and characteristics of the implementation theory, model or framework. Major barriers to selection included inconsistent terminology, poor fit with the implementation context and limited knowledge about and training in existing theories, models and frameworks. Major facilitators to selection included the importance of clear and concise language and evidence that the theory, model or framework was applied in a relevant health setting or context. Participants were enthusiastic about the development of a decision support tool that is user-friendly, accessible and practical. Preferences for tool features included key questions about the implementation intervention or project (e.g., purpose, stage of implementation, intended target for change) and a comprehensive list of relevant theories, models and frameworks to choose from along with a glossary of terms and the contexts in which they were applied.

**Conclusions:**

An easy to use decision support tool that addresses key barriers to selecting an implementation theory, model or framework in practice may be beneficial to individuals who facilitate implementation practice activities. Findings on end-user preferences for tool features and functions will inform tool development and design through a user-centered approach.

## Background

Over 100 different theories, models and frameworks exist to guide effective implementation and sustainability of evidence-based interventions or programs [1, 2]. The myriad of implementation theories, models and frameworks differ in complexity, such as their aim, scope and intended target for change. For example, they may describe the different stages of implementation (e.g., process models); identify barriers and facilitators that influence implementation (e.g., determinant frameworks); or predict or explain implementation success by offering an underlying mechanism or theory of change (e.g., implementation theories) [3]. Further, some theories, models and frameworks are broad and address the entire implementation process, while others focus on a particular implementation aspect such as intervention sustainability. Implementation theories, models and frameworks also operate at one or more levels of change, from a health system to an individual. In many cases, using multiple theories, models and frameworks is useful to inform or address the scope and aims of an implementation project and to guide intervention development and testing at multiple levels [4–6].

Despite a growing interest in the appropriate selection and use of implementation theories, models and frameworks [7–11], it can be difficult to sift through and make sense of the various options available – especially when most are used in practice only once or with limited justification [2, 12]. For instance, participants in an implementation practice training course [13] reported that they struggled to identify and select suitable theories, models or frameworks to guide their work. Studies also suggest that implementation theories, models and frameworks may not be used appropriately [8, 14].

Implementation researchers and practitioners looking to identify a theory, model or framework to inform their work can access existing tools and publicly available resources such as guidance documents (e.g., [15–17]). For example, drawing on their personal experience working with novice implementation practitioners, Lynch and colleagues [10] suggested five questions to consider when selecting a theory, model or framework: who are you working with, when in the process are you going to use theory, why are you applying theory, how will you collect data and what resources are available. Birken and colleagues [9] developed a checklist of 16 criteria (organized within four categories: usability, validity, applicability, acceptability) for implementation researchers or practitioners to consult when selecting a theory, model or framework. A major limitation identified by the tool developers is the prerequisite of a candidate list of suitable theories, models or frameworks to draw from and compare [9]. Rabin and colleagues developed a database of models and frameworks, www.dissemination-implementation.org, however the content is based on the findings of a narrative review of theories, models and frameworks [18] and is not comprehensive.

To address this problem, we propose to use the findings from a rigorous scoping review of over 300 implementation theories, models and frameworks [2] to develop a decision support tool, with input from implementation researchers and practitioners using qualitative research methods. A decision support tool provides structured guidance to help users make an explicit decision [19]. In this case, a decision support tool may facilitate appropriate selection of one or more implementation theories, models or frameworks by engaging the user to answer key questions, resulting in relevant options to consider. The decision support tool will be developed using rigorous methods guided by theory and evidence on user-centered design and implementation science. The overarching approach will be informed using the Knowledge-to-Action Cycle [20] and the United Kingdom Medical Research Council Framework for Development and Evaluation of Complex Interventions [21]. These methods have been used for creation of other decision support tools [22]. As tool development is not the focus for this paper, details on the methods will be described in a subsequent development and evaluation paper.

To inform tool development, we sought the perspectives of implementation researchers and practitioners working in healthcare. Specifically, this study aimed to identify 1) barriers and facilitators to identifying and selecting implementation theories, models and frameworks in research and practice, and 2) preferences for features (i.e., content items) and functions of the proposed decision support tool.

## Methods

Thorne’s interpretive descriptive approach [23] guided all aspects of this research, including the design and analysis. Interpretive description is grounded in traditional qualitative methodologies (e.g., phenomenology) that are derived from the social sciences; yet, it is oriented toward applied health disciplines such as implementation practice and designed to address real-world knowledge gaps [23].

### Study design

We used Thorne’s interpretive descriptive approach to elicit the perspectives of implementation researchers and practitioners through individual interviews. We chose to conduct individual, semi-structured interviews to understand individual perspectives, including challenges and successes related to identifying and selecting implementation theories, models and frameworks in research and practice. While focus groups would have allowed for group interactions and may have helped participants generate and share their ideas [24], we were most interested in individual opinions and decision processes [23]. Therefore, we felt that interviews would be more informative for tool development. Feasibility was also a factor, as our participants were from a wide geographic area. We followed the Consolidated Criteria for Reporting Qualitative Research checklist [25] (Additional file 1). We obtained research ethics board approval from Unity Health Toronto (REB #16–335) and the University of Toronto (REB #33907). Ethics approval covered recruitment at the conferences and workshops, which covered the study participants in the United States (USA) and Australia. Verbal informed consent was approved by the ethics boards and obtained (and audio-recorded) from all participants using a predetermined script prior to the phone interview.

### Participant selection

Eligible study participants included implementation researchers and practitioners (e.g., administrators, clinicians, knowledge brokers) working in healthcare environments such as hospitals, academic research centers or universities, or broader community settings (e.g., public health or regulatory organizations). We defined implementation researchers as individuals who conducted implementation science, and implementation practitioners as individuals who facilitated implementation practice activities (including those who provided support through training and capacity building or knowledge brokering activities).

Study recruitment followed three approaches. First, we recruited in person at two international implementation conferences, one held in the USA in 2016 and one in Canada in 2017. At both conferences, we presented a poster on our scoping review of implementation theories, models and frameworks [2], distributed study information sheets to attendees who stopped to read the poster, and collected contact information from individuals who were interested in participating in our study. We then sent a personalized email to each individual to verify their interest and eligibility and schedule a phone interview. Second, we sent a personalized email to past participants of an implementation practice training course developed by the Knowledge Translation Program (St. Michael’s Hospital, Unity Health Toronto, Canada) [13] and delivered in Canada and Australia between 2015 and 2017. Third, we asked study participants to share the study information sheet with colleagues who might be interested in participating. We sent a personalized email to individuals referred to us by study participants. Up to two more emails were sent to non-responders.

These different recruitment approaches were selected because they targeted diverse implementation researchers and practitioners who were interested in, and had experience with, implementation. The sample was expected to reflect the perspectives of our target end-users of the proposed decision support tool. A sample size of 20–30 participants was expected to provide sufficient information to answer the research question through semi-structured interviews and was considered a feasible range given the available resources [23, 26].

### Data collection

Interviews were conducted over the phone by one investigator (LS) between September 2017 and January 2018. A semi-structured interview guide (Additional file 1) was prepared and revised as needed throughout data collection. Part 1 of the interview explored the barriers and facilitators to identifying, selecting and using implementation theories, models or frameworks in research and practice. It included participants’ views and understanding of theories, models and frameworks and the processes used for considering one or more to inform their implementation activities. The interview guide questions were informed loosely by the Theoretical Domains Framework [27] as a starting point, to allow for inductive analysis. Direct questions inquiring about perceived barriers and facilitators were also included to allow for free-flowing discussion. The Theoretical Domains Framework is a validated determinant framework [28] that has been applied in numerous implementation studies to uncover the underlying barriers to and facilitators of behaviour change. Further, the framework includes a comprehensive set of barriers at the individual or person level, along with the organizational-level (e.g., groups of individuals), which we felt were most important to understand when developing a decision support tool to meet the needs of our targeted end-user. Part 2 of the interview explored the features and functions of a hypothetical decision support tool that would be important to participants as target end-users of the tool. The interview guide was reviewed by and pilot tested with three individuals, all experienced in qualitative research and implementation science and practice, and one of whom was also a clinician. Each interview lasted 30–60 min and was audio-recorded and transcribed verbatim.

### Data analysis

Following an interpretive descriptive approach, we conducted a thematic analysis of the data to synthesize meanings across codes and generate a narrative of the key themes to inform subsequent tool development [23, 29]. Data analysis occurred concurrently with data collection. We used NVivo 12 qualitative data analysis software (QSR International, Cambridge, MA) to organize and code the transcripts. Once the audio-recorded interviews were transcribed and verified for accuracy, they were de-identified using a master linking log, prior to being imported into NVivo. After reading through the first few transcripts to become familiar with the data, we used open coding to create codes from the text and drafted a coding framework. This coding framework was revised iteratively throughout data collection and analysis. All data were coded inductively by a single investigator (LS), with a subset of 20% (i.e., 5 transcripts in total) coded by a second investigator (JB) with high concordance achieved. This duplicate coding process was done at the start and end of data collection to ensure consistency of themes. Representative quotes from participants were selected to support the themes and study findings. The final manuscript was shared with participants for feedback on the research findings.

## Results

### Participant characteristics

Twenty-four individuals consented to participate: 16 were from Canada, seven from the USA and one from Australia (Table 1). One eligible participant declined consent due to a confidentiality agreement with their current employer. Of the eligible workshop participants contacted, 2 were not reached due to undeliverable email addresses and 33 did not respond to our email invitation. Participants were recruited until no new themes were identified; therefore, not all workshop participants were sent a study invitation. Participants worked in a variety of healthcare environments including hospitals, academic research centers, universities, government organizations, and regulatory organizations. Participants had a range of experience supporting implementation activities in healthcare environments and reported working in implementation for 1.5 to over 20 years. Of the 24 participants, 11 (46%) had completed a “Practicing Knowledge Translation” course developed by the Knowledge Translation Program at St. Michael’s Hospital, Unity Health Toronto, Canada [13]. In terms of knowledge, 14 (58%) participants rated themselves as very or extremely knowledgeable or familiar with implementation theories, models and frameworks, and 13 (54%) as very or extremely confident in selecting and applying them to their work. Sixteen (67%) participants reported frequently or always selecting an implementation theory, model or framework and applying it to their work.

**Table 1:** Participant characteristics

Participant characteristics (*n* = 24)	n	%
Geographic location		
Canada	16	67
USA	7	29
Australia	1	4
Type of healthcare environment*		
Healthcare organization	17	71
University	15	63
Funding or regulatory organization	4	17
Years of implementation experience in healthcare environment		
1–2 years	5	21
3–5 years	5	21
6–10 years	3	13
More than 10 years	11	46
Past “Practicing Knowledge Translation” workshop participant^		
Yes	11	46
No	13	54
Level of knowledge selecting and applying implementation theory, model or framework		
Not at all knowledgeable	0	0
Slightly knowledgeable	5	21
Neutral	5	21
Very knowledgeable	12	50
Extremely knowledgeable	2	8
Level of confidence selecting and applying implementation theory, model or framework		
Not at all confident	0	0
Slightly confident	2	8
Neutral	9	38
Very confident	10	42
Extremely confident	3	13
Frequency of selecting and applying implementation theory, model or framework		
Never/not applicable now	2	8
Rarely	2	8
Sometimes	4	17
Frequently	9	38
Always	7	29

*Not mutually exclusive

^Workshop (Moore et al., [13]) delivered at either St. Michael’s Hospital, Canada or Bond University, Australia

### Barriers and facilitators to identifying and selecting implementation theories, models or frameworks

**Fig. 1:**
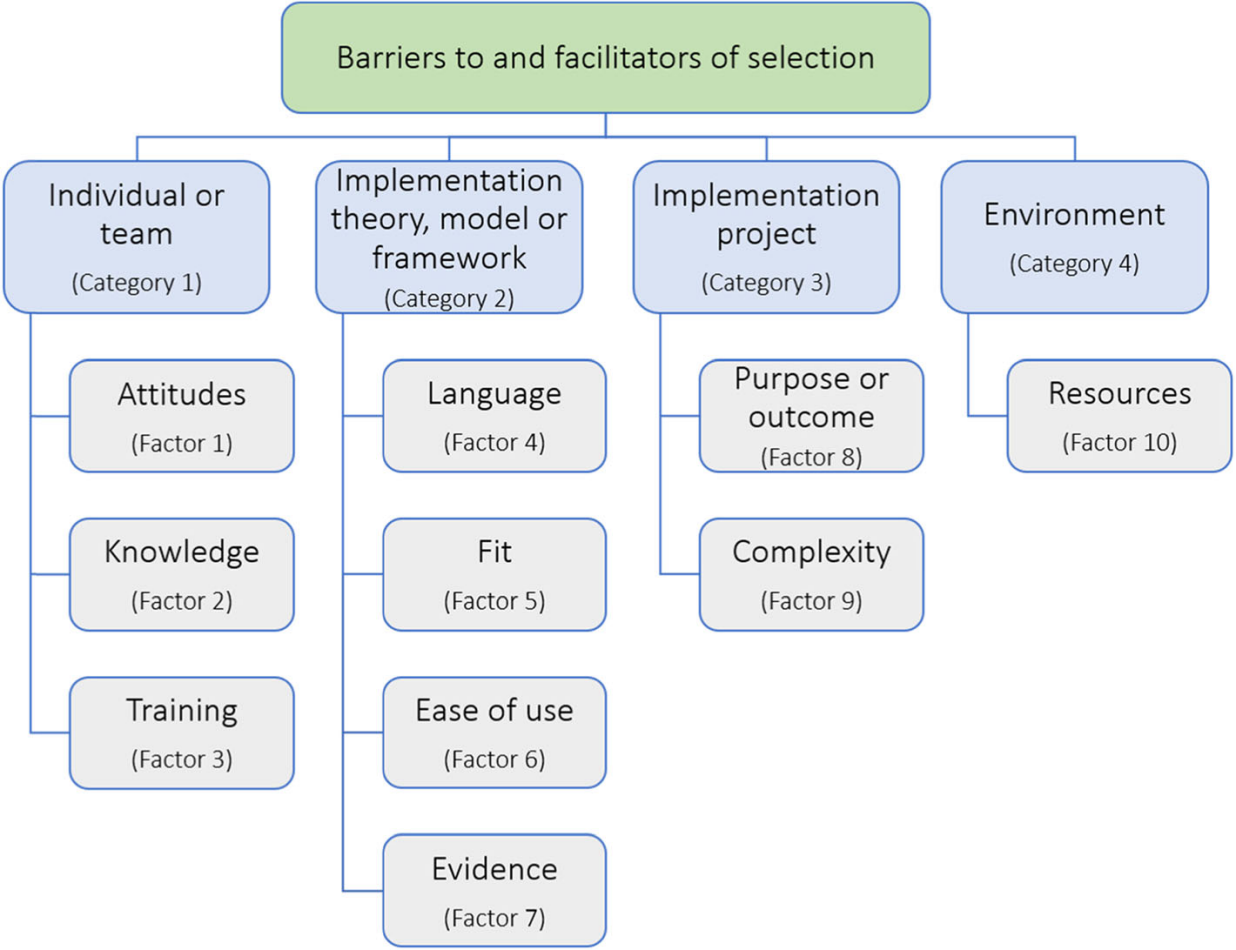
Categories and factors influencing the identification and selection of an implementation theory, model or framework

Four broad categories and 10 factors, generated from the data, influenced identification and selection of implementation theories, models and frameworks and were relevant to tool development (Fig. 1). Illustrative interview excerpts are presented in Tables 2 and 3.

**Table 2:** Interview excerpts supporting key factors related to category 1 ‘characteristics of individual or team conducting implementation’

Interview excerpts reflective of factor 1 ‘attitudes’:	
“*… sometimes the people you work with don’t always appreciate trying to stay true to a theory, model or framework or not always understand the value of it and how there are methods to KT [knowledge translation] and it’s not something that we just do on a whim.” (ID22)* *“What I am appreciating more and more, is that there needs to be a theoretical basis for a lot of the things that we do … and it doesn’t have to be one size fits all because there are different models and frameworks and theories applied to different interventions or problems.” (ID8)*	
Interview excerpts reflective of factor 2 ‘knowledge’:	
*“You sort of get familiar enough with certain frameworks, and then it’s just much easier to write about them and it’s much easier to build upon your previous work.” (ID2)* “*… if the only way that they can kind of access and engage with these frameworks is by going into large voluminous quantities of the research literature, then there is a disincentive to use it.” (ID11)*	
Interview excerpts reflective of factor 3 ‘training’:	
*“I enjoy the TDF [Theoretical Domains Framework] and the Knowledge-to-Action Cycle because I’ve been to two workshops on it by people who use it regularly and have written about it, so you get a much better understanding and you can play around with the concepts before you go deep into a research project. Whereas with Normalization Process Theory, I haven’t had any instruction on it and that might make me feel quite differently about it.” (ID6)* *“I don’t know if I know anyone who’s working in KT [Knowledge Translation] that has more than a week or two of formal training to do with theories, concepts, strategies and what’s evidence-based and that really delves into all aspects of KT.” (ID21)* *“Healthcare practitioners aren’t necessarily given education on theories of change. So, when they’re asked to adopt or to look more deeply into a theory of behaviour change … they don’t consider themselves to have enough expertise to use them.” (ID24)*

**Table 3:** Interview excerpts supporting key factors related to category 2 ‘characteristics of implementation theory, model or framework’

Interview excerpt reflective of factor 4 ‘language’:	
*“Some of the language that we use is kind of common language and so you might think organizational readiness for change for instance, it’s easy to think ‘oh we kind of know what that means’ and so then, when we don’t explicitly define it … it presents a challenge for the field.” (ID2)*	
Interview excerpts reflective of factor 5 ‘fit’:	
*“What are the particular circumstances under which the Quality Improvement Implementation Framework with its 14 steps is better than the Interactive Systems Framework which has essentially three interacting domains? What is the circumstance under which I might want to use the Theoretical Domains Framework compared to using CFIR [Consolidated Framework for Implementation Research] for example? And what are the kinds of practical problems where one would work relative to the other?” (ID11)* *“I think having practical examples and having models, frameworks that have been used for real practice changes and having those examples that you can then, you know, see how they were applied, that is helpful. And then, that also helps to refine those theories and models and frameworks into something that is very application-oriented and works.” (ID15)*	
Interview excerpts reflective of factor 6 ‘ease of use’:	
*“It’s almost like they [theories, models and frameworks] need their own form of translation.” (ID6)* *“I know there’s a lot of different theories and frameworks that operate at various levels. So, you have those very high level, broad applicable theories that … can be quite challenging in how to drill down and apply them in an operational setting. But there are also some really focused local level theories that you can use to guide the work.” (ID8)* *“As you look at each domain, how are you going to measure it and does that measurement approach, whether it’s quantitative or qualitative, make sense for the context or the populations you’re working with.” (ID4)*	
Interview excerpt reflective of factor 7 ‘evidence’:	
*“It would be nice [to] summarize the evidence underpinning the different models, right? And by that, I mean not only the evidence used to create the models. Probably what would be even better is if there was evidence to suggest that the models actually facilitate or improve knowledge translation. That would be great.” (ID9)*	

#### Category 1: characteristics of the individual or team conducting implementation

##### Factor 1: attitudes about the importance of selecting theories, models and frameworks

Participants reported having a general understanding of theories, models and frameworks and described several uses in implementation research and practice. For example, many participants found Nilsen’s 2015 taxonomy [3] was useful for defining a theory versus a model versus a framework and referred to the taxonomy when describing their similarities and differences. Some participants said their understanding was grounded in their learnings from the “Practicing Knowledge Translation” course. Others described their understanding of implementation theories, models and frameworks in terms of their clinical or health discipline, such as the Iowa Model for Evidence-based Practice to Promote Quality Care [30] which originates in the nursing field. In general, frameworks and models were described as being descriptive and useful for clarifying aspects of a complicated process. Theories were viewed as being more explicit about how certain phenomena are operating and how change might be occurring.

Participants mentioned using 28 different implementation theories, models and frameworks to inform their work (Table 4). Participants described the important role that theories, models and frameworks play in advancing implementation understanding, especially regarding planning, developing and sustaining effective interventions and implementation strategies. Some of the described uses of theories, models and frameworks included: informing the research question; justifying and organizing an implementation project; guiding the selection and tailoring of implementation strategies; helping to achieve intended outcomes; and analyzing, interpreting, generalizing, or applying the findings of an implementation project. Other benefits to their use included providing a good starting point for implementation, providing a systematic or pragmatic approach for implementation, avoiding overlooking key categories or processes of implementation, and increasing methodological rigor. Participants commented on the importance of engaging in practices that are informed by theories, models and frameworks and evidence.

**Table 4:** Implementation theories, models and frameworks used by participants

Active Implementation Framework	
Behaviour Change Wheel	
Capability Opportunity Motivation Behavior	
Consolidated Framework for Implementation Research	
Diffusion of Innovation	
Diffusion of Innovations in Health Service Organizations	
Exploration, Preparation, Implementation and Sustainment Model	
Grol and Wensing’s Model for Effective Implementation	
Interactive Systems Framework	
IOWA Model of Evidence-based Practice	
Kern’s Medical Model for Curriculum Development	
Knowledge-to-Action Framework	
Lavis’ Framework for Knowledge Transfer	
Lewin’s Change Theory	
NHS Sustainability Model	
Normalization Process Theory	
Plan-Do-Study-Act Cycles	
Practical Robust Implementation and Sustainability Model	
Proctor’s Implementation Outcome Framework	
Promoting Action on Research Implementation in Health Services	
Quality Implementation Framework	
QUERI Model	
Reach Effectiveness Adoption Implementation Maintenance	
Replicating Effective Programs Model	
Social Cognitive Theory	
Star Model of Knowledge Transformation	
Theoretical Domains Framework	
Transtheoretical Model of Behaviour Change	

Note: nearly half of the 24 participants attended the same training course, which may have limited the range of theories, models and frameworks identified. See Additional file 1 for citations for the theories, models and frameworks.

While all participants agreed on the utility of frameworks and models, such as the Knowledge-to-Action Cycle [20], a few were skeptical of the value of using theory to enhance knowledge of the complexity of implementation; they preferred to avoid selecting a formal theoretical approach. Others lacked experience with theory-driven implementation. A few believed that implementation practitioners may not feel the same level of “pressure” to use a theory, model or framework in their role compared to an implementation researcher.

##### Factor 2: knowledge of existing implementation theories, models and frameworks

Knowledge of existing implementation theories, models and frameworks and where to find them were perceived to be important. Some participants struggled to identify new theories, models or frameworks to inform their work, and identified their lack of knowledge of the breadth of options as an important barrier. Most participants favoured one or more implementation theories, models or frameworks and used them repeatedly, stating that it was easy to use what was familiar. Many did not follow an explicit process for identifying a new theory, model or framework. Access to a comprehensive repository or database of existing implementation theories, models and frameworks was perceived as helpful. Participants also suggested having at least one implementation team member with up-to-date knowledge of what implementation theories, models and frameworks exist, where to find them and their uses.

##### Factor 3: training related to implementation theories, models and frameworks

Participants talked about the relationship between selecting implementation theories, models and frameworks in research or practice and their training experience. For example, most participants selected theories, models and frameworks for which they received specific training. Major barriers to selection included inadequate background or research training in implementation theories, models and frameworks, and lack of training or expertise in implementation research methods or practice. Some participants spoke about the challenge of getting others (e.g., senior administrators, healthcare providers) to buy into the use of a certain theory, model or framework, especially if they were not familiar with the application of theory. Facilitators to selection included gaining appropriate training through participation in capacity building activities, such as accessing implementation workshops, conferences, coaching, mentoring, train-the-trainer approaches or communities of practice. Examples included working with someone who was formally trained on the theory, model or framework, or receiving feedback from implementation experts who used it to inform their work.

#### Category 2: characteristics of the implementation theory, model or framework

##### Factor 4: language and terminology used to describe the theory, model or framework

Language and terminology were key factors for identification and selection. Participants described the language used in implementation theories, models and frameworks as “complex”, “abstract”, “complicated” and “confusing”. In particular, the use of jargon and lack of clear construct definitions were identified as major barriers. Further, several participants struggled with overlapping constructs, and the inconsistent terms used to describe them across theories, models and frameworks. For example, the same term or definition may be used for different constructs, or different terms or definitions may be used for the same constructs. A few participants commented on the inaccurate and inconsistent use of the term theory versus model versus framework, both in research and in practice settings. This appeared to be common with theories versus frameworks (e.g., calling something a theory but referring to a framework). Facilitators included the importance of clear and concise language, and clearly-defined constructs to help differentiate among the various theories, models and frameworks.

##### Factor 5: fit of the theory, model or framework to the implementation project

Another key factor for identification and selection was the level of fit or appropriateness of the theory, model or framework to the implementation project. Specifically, a poor fit between the context in which the theory, model or framework was developed or had been applied, and the context of the implementation project was identified as a major barrier. For example, many theories, models and frameworks were developed for a specific condition or health behaviour and had not yet been applied in different contexts. Important aspects of the context included the research question, purpose or goal; health problem; setting; population; and level of behaviour change. Evidence that the theory, model or framework had been applied in practice in a similar context (such as relevant examples of applications in the literature) facilitated appropriate selection. Participants stated that seeing a description of the contexts in which the theory, model or framework was previously used was helpful when determining fit. Being aware of a theory, model or framework’s underlying assumptions and its limitations also informed appropriateness and applicability. Other related challenges included the interchangeability, compatibility and adaptability of implementation theories, models and frameworks. For example, some participants struggled with the trade-offs of selecting one theory, model or framework over another. Participants perceived that guidance on comparing different options would facilitate appropriate selection. Some noted that theories, models and frameworks often overlap or are highly derivative of each other, which adds to the complexity of combining more than one within an implementation project. It was deemed helpful to highlight theories, models or frameworks that fit well together, such as the research by Michie and colleagues linking Capabilities Opportunities Motivation Behaviour with the Theoretical Domains Framework [31]. For others, implementation theories, models or frameworks that allowed for some modification were appealing, but participants struggled with how to modify or change aspects to improve fit while maintaining fidelity to key elements.

##### Factor 6: ease of use of the implementation theory, model or framework

Ease of use in practice was perceived to influence selection of a theory, model or framework. Some participants described implementation theories, models and frameworks as “not intuitive to use” and difficult to operationalize in the context of their own implementation project, even when the theory, model or framework was viewed as a relevant option. Facilitators to selection and use included existing online tools and publicly available resources, such as websites dedicated to specific theories, models or frameworks (e.g., the Consolidated Framework for Implementation Research). In terms of measurement challenges, a few participants cited a lack of relevant measures for key variables across theories, models and frameworks, as well as variability in the extent to which measures were developed to assess constructs. Participants preferred theories, models or frameworks that were “highly actionable”, “pragmatic” and “easy to operationalize” in practice, with detailed processes for the measures themselves that were compatible with their setting.

##### Factor 7: evidence supporting the implementation theory, model or framework

Empirical evidence of effectiveness, including strength of evidence supporting the theory, model or framework, influenced selection. Implementation theories were described as “fairly loose” and “without solid evidence” compared to theories in other scientific fields (e.g., physical sciences). Further, within a theory, model or framework, the level of evidence was perceived to be uneven across domains or specific processes. A summary of the evidence supporting a theory, model or framework, including the evidence used to create it and evidence of its effectiveness, was deemed to be an important facilitator. Participants also felt it was important that the theory, model or framework constructs and concepts had face validity and made sense in terms of the implementation research question or goal.

### Categories 3 and 4

Other important barriers and facilitators mentioned by participants were related to characteristics of the healthcare environment (Category 4) and, to a lesser extent, characteristics of the implementation intervention or project (Category 3).

Availability of resources (Factor 10) within complex healthcare environments (Category 4), such as time, staffing and capacity, funding and access to data were identified as both barriers and facilitators to selection. Many participants also described a “tension” between time and robustness of implementation. For example, a lack of time to invest in the understanding and use of a theory, model or framework (e.g., competing demands or pressure to fix the problem right away) was a major barrier, while taking the time to create an implementation plan that included consideration of theories, models or frameworks at implementation onset was a facilitator. Theory, model or framework selection was also influenced by staff and stakeholder support, such as having an inadequate number of project staff available or being the sole implementation practitioner within an organization. It was deemed important to “assemble the right people at the right table” to avoid siloed practice and redundancy.

Finally, a few participants mentioned factors related to the implementation project (Category 3), such as consideration of the purpose, problem or goal and intended outcome (Factor 8). For instance, it may be inappropriate to select a theory when part of the research question or outcome of an implementation project was to further develop theory. Another relevant factor that presented a challenge to selection was the level of intervention complexity (Factor 9), including the type of intended behaviour change (e.g., individual, program, practice, policy), and the implementation stage (e.g., planning, evaluation, sustainability) for the project.

### Features and functions of a decision support tool

Participants were enthusiastic and receptive to the idea of a decision support tool targeted to implementation practitioners. The following key features and functions were suggested to inform tool development. Illustrative interview excerpts are presented in Table 5.

**Table 5:** Interview excerpts supporting key tool features and functions

Interview excerpts reflective of suggested tool features:	
*“You shouldn’t be leading in from the framework itself, you should be leading in from the kinds of problems the framework solves.” (ID11)* *“You really have to think about … what is the purpose of the KT [knowledge translation] activity and what level you’re trying to implement, or trying to facilitate knowledge translation to occur, because not all models, theories and frameworks will fit, right?” (ID3)* *“Why I always start with the Knowledge-to-Action Cycle is because it helps me break it down. I know where to apply different theories within that framework...So, I think you have to break down the important components of doing KT [knowledge translation] and making it very clear.” (ID13)* *“Being able to see the extent of what the possibilities and the options are and what kind of context they’ve been used before and what purpose … and examples I guess of what’s been done with those, like actual practical real-life examples of what was done with those theories, models and frameworks would be helpful.” (ID17)*	
Interview excerpts reflective of suggested tool functions:	
“*… easy, accessible and not too many clicks and not too many words.” (ID12)* *“I think the idea of creating a decision tool is great, but just from my work with people, it’s that constant tension between having it be robust enough to do what it’s supposed to do, but simple enough so that people don’t glaze over,” (ID18)* *“Filters – so asking people to identify who their audience is for the implementation, what the context is and any other kind of relevant features and then having a list of potential theories, tools and models that they can employ in their implementation, so kind of narrowing down the scope.” (ID24)* *“There may be a need for a decision support tool that has multiple levels to it or depths in some way, so there could be a superficial identification of candidate models for use or for consideration. And then one could look and scan to determine which ones makes sense and then dive deeper into how one of those models might be used or elements of the model that can be pulled out.” (ID9)* *“I like that it’s online … any consultation I do or anywhere I go, it’s very easy to access.” (ID25)*	

#### Features or content items

Most importantly, the tool should include a comprehensive list of existing implementation theories, models and frameworks to choose from. Suggested content items included characteristics of the theories, models and frameworks matched with characteristics of the end-user’s implementation project (e.g., aim, scope and level of change). Participants suggested organizing the theories, models and frameworks according to their purpose (including their intended aim, scope and level of change) to align them with end-users’ needs. Alternatively, one participant (ID1) suggested starting with the project end goal or outcome, and reviewing theories, models and frameworks that include that outcome as a relevant construct. Many participants also suggested including the context in which the theories, models and frameworks have been applied, along with links to seminal articles and examples of real-world use. Linking the tool with seminal articles would allow end-users to see examples of what has been done, and perhaps gauge ease of use, as well as where the literature may or may not be saturated. Some participants suggested summarizing the evidence supporting each theory, model and framework to highlight those that have been validated. A few participants suggested content items related to the availability of implementation resources, such as the project timelines, number of stakeholders, guidance and team expertise, and financial support.

#### Functions

Participants suggested that the tool be simple and easy to use by the target end-user (i.e., implementation practitioners). They identified that it should provide the user with a modest set of key questions or prompts that start off broad and become more specific. For example, the tool could respond to the user’s input by guiding them toward more specific theories, models and/or frameworks. The tool should also be practical in that the level of content detail fits the intended tool audience and purpose. Being highly accessible through an open access web-based platform was also important. Further, accommodating a team-based approach (e.g., permitting access and use of the tool by an entire multi-disciplinary implementation team) would foster collaboration. Other suggested features included: interactive viewing or search capabilities (e.g., clicking on an interactive theory, model or framework diagram or figure for more information, or searching by key word or construct name); webinars or instructional videos led by experts on when (and how) to use the theory, model or framework; the use of “storytelling” (e.g., case studies) to increase personal connection; and built-in chat room capabilities to connect or collaborate with and receive feedback from others in the field who have experience selecting and using the implementation theory, model or framework. Finally, a few participants suggested an embedded evaluation component whereby users may consent to complete a survey to provide feedback on the tool.

## Discussion

Our findings revealed that factors related to the theory, model or framework, the individual or team conducting implementation and the implementation project are critical to consider when developing a decision support tool. Key barriers to selection related to characteristics of the theory, model or framework included: inaccurate and inconsistent language, poor fit with the implementation context, lack of appropriate measures and limited empirical evidence of effectiveness. These findings are supported by a recent, international survey of over 200 implementation researchers and practitioners who rated ‘empirical support’ and ‘application to a specific population or setting’ as the most important criteria for selection; nevertheless, survey respondents also reported selecting a theory, model or framework based on convenience or familiarity [1]. Similarly, we found that a lack of knowledge of and familiarity with existing implementation theories, models and frameworks, along with a lack of proper training on their use, were key individual/team-level barriers to selection. These knowledge and skills barriers were not surprising given the abundance of implementation theories, models and frameworks coupled with low citation rates in the literature, indicating they are not commonly used [2, 12]. Our study reaffirmed this finding by demonstrating that a group of implementation researchers and practitioners with high self-rated knowledge and experience generated a list of 28 theories, models, and frameworks, which represent less than 20% of those identified in a scoping review. While there may be benefits to selecting a highly-cited theory, model or framework (such as comparability of results across populations or health behaviours [32] or greater availability of resources for operationalization and measurement [12]), a systematic and comprehensive approach to theory, model and framework identification and selection is necessary to advance implementation science and practice.

There are numerous determinant frameworks that we could have chosen to inform our interview guide. For example, our team recently mapped over 300 implementation theories, models and frameworks to Nilsen’s taxonomy [3] and identified over 50 determinant frameworks targeting at least individual-level change; however, many did not include a comprehensive set of barriers and facilitators (*unpublished data*). Our findings on the barriers and facilitators to selection of a theory, model or framework, in the context of informing a decision support tool, are supported by the Theoretical Domains Framework. For example, the domain ‘knowledge’ considers having the knowledge to locate and understand existing theories, models and frameworks. The ‘skills’ and ‘beliefs about capabilities’ domains focus on having the skills required to know how to select a theory, model or framework in practice and considers how easy or difficult this task is for an individual or team. The ‘social/professional role and identity’ and ‘optimism’ domains consider attitudes about the importance of using theories, models and frameworks, specifically whether an individual believes that selecting and using them is part of their role as an implementation researcher or practitioner and that doing so will benefit their implementation work. The ‘goals’ and ‘intentions’ domains focus on wanting to select and use theories, models and frameworks and then making a conscious decision to include them in implementation work, for example, by using a decision support tool. Finally, the ‘environmental resources’ domain considers having the time and funds to invest in the selection process.

A decision support tool addressing our findings on barriers and facilitators to selection might include a comprehensive list of theories, models and frameworks, a glossary of key terms, the contexts in which the theories, models and frameworks have been developed and applied (including examples of application), and any available evidence to support their validity. Other suggested features for consideration during tool development included the purpose, goal or intended outcome of the implementation project as well as the target population and the intended target for change. It would be quite challenging as tool developers, to systematically categorize existing theories, models and frameworks according to factors such as the amount of time or funding required for use; it may be more beneficial for end-users to reflect on these environment-level factors as key considerations associated with the selection of a particular theory, model or framework from the options provided by the tool. Findings on end-user preferences for tool features and functions will inform tool development and design through a user-centered approach [33].

### Limitations

The following study limitations should be considered. First, we used a convenience sample of implementation conference and course attendees. As a result, close to half of our participant sample completed a “Practicing Knowledge Translation” course. As such, we were mindful during recruitment to ensure representatives from different types of healthcare environments, roles, and level of experience. Although we did not intend to saturate these fields given our sample size, we did obtain saturation of themes and had a good sample size for qualitative interviews [23]. Second, we chose to interview implementation researchers and practitioners with some implementation practice experience (i.e., as the target end-users of our tool) because we felt that this experience would be necessary to identify the underlying barriers and facilitators. As such, all study participants described having a baseline understanding of at least a few implementation theories, models and frameworks. While many participants rated their knowledge and confidence with identifying, selecting and using implementation theories, models and frameworks as fairly high, for many this rating reflected their knowledge and confidence regarding the theories, models or frameworks that they were most familiar with and used repeatedly to guide their work.

## Conclusion

Individuals who are doing implementation work face many challenges, including how to identify and select appropriate implementation theories, models and frameworks to inform their projects. Key barriers to selection identified in this study included inconsistent language, poor fit and limited knowledge about and training in theories, models and frameworks. These barriers, together with the findings of our scoping review on existing theories, models and frameworks, will inform and tailor the features and functions of a proposed decision support tool for use by implementation practitioners. Our findings from this interview-based study suggest the tool should be easy to use, accessible and feature questions about the implementation project’s purpose, scope and intended target for change, in addition to presenting a comprehensive list of relevant theories, models and frameworks and the contexts in which they were applied.

## Supplementary information

**Additional file 1.** Supplemental file with COREQ checklist (Appendix 1), interview guide (Appendix 2), and citations for theories, models and frameworks used by participants (Appendix 3).

## Data Availability

Not applicable.
