# Supplementary material for: Identifying and selecting implementation theories, models and frameworks: a qualitative study to inform the development of a decision support tool
Source: BMC Med Inform Decis Mak. 2020 May 14;20:91. doi: 10.1186/s12911-020-01128-8 (PMC7227323; doi:10.1186/s12911-020-01128-8)
Supplement: Supplementary file 1 — Additional file 1. Supplemental file with COREQ checklist (Appendix 1), interview guide (Appendix 2), and citations for theories, models and frameworks used by participants (Appendix 3). [file 12911_2020_1128_MOESM1_ESM.pdf]

SUPPLEMENTAL FILE: Identifying and selecting implementation theories, models and frameworks: a qualitative study to inform the development of a decision support tool

Contents

Appendix 1. Consolidated criteria for reporting qualitative studies (COREQ) 2

Appendix 2. Telephone interview guide for implementation researchers and practitioners 5

Appendix 3. Citations for 28 implementation theories, models and frameworks used by participants and listed in Table 4 7

Appendix 1. Consolidated criteria for reporting qualitative studies (COREQ)

Item	Description
**Domain 1: Research team and reflexivity**	
1. Interviewer/facilitator	Lisa Strifler
2. Credentials	BSc, MSc, PhD Candidate
3. Occupation	PhD Student
4. Gender	Female
5. Experience and training	Lisa Strifler is a PhD candidate who conducted this research as part of her PhD thesis project. She has training in health services research and methods and received formal training in qualitative research methodology during her graduate studies.
6. Relationship established	Email communication was exchanged between Lisa Strifler and the participant to determine eligibility and schedule an interview.
7. Participant knowledge of the interviewer	Participants were aware of the purpose and rationale of the study, that it was being conducted as part of Lisa Strifler's PhD thesis project, and the project funding source.
8. Interviewer characteristics	Lisa Strifler disclosed her role as a PhD candidate at the University of Toronto, a graduate trainee with the Knowledge Translation Program at St. Michael's Hospital in Toronto, Canada, and primary researcher on this project.
**Domain 2: study design**	
9. Methodological orientation and theory	Interpretive description and thematic analysis
10. Sampling	Convenience sampling was used, while being mindful during recruitment to ensure representatives from different types of healthcare environments, roles and level of experience.
11. Method of approach	Conference attendees were recruited in person and via personalized email. Workshop participants were recruited via personalized email.
12. Sample size	24 participants. A target sample size of 20-30 participants was expected to provide sufficient information to answer the research question through semi-structured interviews and was considered a feasible range given the available resources.
13. Non-participation	1 eligible participant declined consent due to a confidentiality agreement with their current employer; 2 eligible participants were not reached due to undeliverable email addresses; 33 eligible workshop participants did not respond to our email invitation. Participants were recruited until no new themes were identified; therefore, not all workshop participants were contacted/invited to participate.
14. Setting of data collection	Phone interviews were conducted from Lisa Strifler's office at St. Michael's Hospital or from her home. Participants participated from their preferred location.
15. Presence of non-participants	A qualitative methodologist listened in on the first interview to provide guidance and feedback to Lisa Strifler once the interview

	was complete. The participant was informed of the methodologist's name and role prior to the start of the interview. The participant provided their consent to have the methodologist listen on the line. No other non-participants were present during the interviews.
16. Description of sample	See Table 1.<br>A majority of participants were from Canada. Participants worked in a variety of healthcare environments and had a range of experience supporting implementation activities in healthcare environments. Participants had high self-rated knowledge and experience using certain implementation theories, models and frameworks that were familiar to them.
17. Interview guide	See Appendix 2.<br>A semi-structured interview guide was prepared and revised as needed throughout data collection. The interview guide was reviewed by and pilot tested with 3 individuals who had experience with qualitative research and implementation science and practice, of which one was also a clinician.
18. Repeat interviews	Repeat interviews were not carried out.
19. Audio/visual recording	Interviews were audio-recorded and transcribed verbatim.
20. Field notes	Lisa Strifler took notes during and immediately following each interview and referred to these notes during data analysis and interpretation.
21. Duration	Interviews lasted 30 to 60 minutes.
22. Data saturation	Participants were recruited until no new themes were identified.
23. Transcripts returned	We did not return individual transcripts to participants for comment. However, the draft manuscript was shared with all participants for feedback on the research findings.
**Domain 3: analysis and findings**	
24. Number of data coders	Data were inductively coded by a single investigator with a subset of 20% coded independently by a second investigator.
25. Description of the coding tree	A description of the coding tree has not been provided but will be made available upon request.
26. Derivation of themes	Themes were derived from the data.
27. Software	NVivo 12 qualitative data analysis software (QSR International, Cambridge, MA) was used to organize and code the transcripts.
28. Participant checking	The final draft manuscript was shared with all participants for their feedback.
29. Quotations presented	See Tables 2, 3 and 5.<br>Direct quotes from participants were presented in tables to support the themes and study findings. Each quotation was identified using participant ID numbers.
30. Data and findings consistent	The data and findings are consistent.
31. Clarity of major themes	See Figure 1.<br>Major themes underlying the perceived barriers and facilitators

	to selecting theories, models and/or frameworks included: (1) characteristics of the individual or team conducting implementation, including their attitudes, knowledge and training; (2) characteristics of the implementation theory, model or framework, including language, fit, ease of use and empirical evidence; (3) characteristics of the implementation project, including the purpose or outcome and level of complexity; and (4) characteristics of the environment, specifically availability of resources. The discussion places these findings in context with other literature showing a lack of use of theories, models and frameworks in practice.
32. Clarity of minor themes	See Figure 1.<br>Major themes were further categorised as minor themes, for example (1) characteristics of the individual or team conducting implementation was further categorized as (a) attitudes, (b) knowledge and (c) training.

Appendix 2. Telephone interview guide for implementation researchers and practitioners

Study ID: ________

Role and experience

1. Could you please describe your healthcare organisation or environment and your current role supporting knowledge translation (KT)/Dissemination & Implementation (D&I) activities within that environment?

2. Approximately how many years of experience do you have supporting KT/D&I activities in a healthcare organisation or environment?

Understanding and conceptualization of theories, models and frameworks

3. Could you spend a few minutes describing your general understanding of KT/D&I theories, models and/or frameworks? For example, how you define or view them, and your familiarity with them?

4. What is your level of knowledge in terms of identifying, selecting and applying theories, models or frameworks to KT/D&I activities in practice? (1-Not at all knowledgeable, 2-Slightly knowledgeable, 3-Neutral, 4-Very knowledgeable, 5-Extremely knowledgeable)

5. What is your level of confidence in your ability to identify, select and apply KT/D&I theories, models or frameworks in practice? (1-Not at all confident, 2-Slightly confident, 3-Neutral, 4-Very confident, 5-Extremely confident)

6. What is your frequency of identifying, selecting and applying theories, models or frameworks to KT/D&I activities in practice? (1-Never, 2-Rarely, 3-Sometimes, 4-Frequently, 5-Always)

Use of theories, models and frameworks

7. In general, what processes or approaches do you use (or have you used in the past) for identifying and selecting theories, models or frameworks to inform your KT/D&I activities (if any)?
   a. What challenges have you experienced with identifying, selecting and applying KT/D&I theories/models/frameworks (if any)?
   b. What have been the successes (if any)?

8. Now I'd like you to think about how you've used a theory, model or framework in a specific KT/D&I project. How did you go about selecting an appropriate KT/D&I theory, model or framework? [If did NOT use a KT/D&I theory, model or framework to inform work, why not?]
   a. Was it easy or difficult for you to identify one? How so?

b. In your view, what made the theory, model or framework appropriate to use in that particular case?

Barriers and facilitators

9. Overall, what do you think are the greatest challenges to identifying, selecting and using theories, models or frameworks in practice when implementing interventions in health care?
   a. How and why are these factors major issues or barriers to success?
   b. How do you think they could be addressed?

10. What factors do you think facilitate the identification, selection and use of KT/D&I theories, models or frameworks in practice?
    a. How and why are these factors facilitators to success?

Features and functions of tool

11. Some individuals in the field feel there is potential for a decision support tool to try to enhance appropriate selection and use of theories, models and frameworks. If you would now imagine that you are doing an implementation project and are looking to identify a KT/D&I theory, model or framework to inform the project, which features (or content items) would be useful for a decision support tool to have, in order to facilitate this task? Why?

12. In addition to features or content items, are there any aspects of the functionality of the tool that come to mind as being potentially useful? Why?

Additional comments and wrap-up

13. Before we wrap up, do you have any additional comments to add regarding the selection and use of KT/D&I theories, models or frameworks?

14. Thank you! If you think of anyone else who might be interested in participating in a similar interview, please feel free to share the study information sheet with them, which contains my contact information.

Appendix 3. Citations for 28 implementation theories, models and frameworks used by participants and listed in Table 4

Name:	**Active Implementation Framework**
Citation:	Fixsen DL, Naoom SF, Blase KA, Friedman RM, Wallace F. Implementation research: a synthesis of the literature. Tampa FL: University of South Florida, Louis de la Parte Florida Mental Health Institute, The National Implementation Research Network, 2005 (FMHI Publ. #231).
Website:	https://nirn.fpg.unc.edu/ai-hub
Name:	**Behaviour Change Wheel**
Citation:	Michie S, van Stralen MM, West R. The behaviour change wheel: a new method for characterising and designing behaviour change interventions. Implement Sci 2011;6:42.
Website:	http://www.behaviourchangewheel.com/about-wheel
Name:	**Capability Opportunity Motivation Behavior (COM-B)**
Citation:	Michie S, van Stralen MM, West R. The behaviour change wheel: a new method for characterising and designing behaviour change interventions. Implement Sci 2011;6:42.
Website:	http://www.behaviourchangewheel.com/about-wheel
Name:	**Consolidated Framework for Implementation Research (CFIR)**
Citation:	Damschroder LJ1, Aron DC, Keith RE, Kirsh SR, Alexander JA, Lowery JC. Fostering implementation of health services research findings into practice: a consolidated framework for advancing implementation science. Implement Sci 2009;4:50.
Website:	http://cfirguide.org/
Name:	**Diffusion of Innovation**
Citation:	Rogers EM. Diffusion of innovations. 5th ed. New York: Free Press; 2003.
Website:	Not applicable
Name:	**Diffusion of Innovations in Health Service Organizations**
Citation:	Greenhalgh T, Robert G, Macfarlane F, Bate P, Kyriakidou O. Diffusion of innovations in service organizations: systematic review and recommendations. Milbank Q 2004;82(4):581-629.
Website:	Not applicable
Name:	**Exploration, Preparation, Implementation, Sustainment (EPIS) Framework**
Citation:	Aarons GA, Hurlburt M, Horwitz SM. Advancing a conceptual model of evidence-based practice implementation in public service sectors. Adm Policy Ment Health 2011;38(1):4-23.
Website:	https://episframework.com/
Name:	**Grol and Wensing's Model for Effective Implementation**
Citation:	Grol R, Wensing M. What drives change? Barriers to and incentives for achieving evidence-based practice. Med J Aust 2004;180:S57-60.
Website:	Not applicable
Name:	**Interactive Systems Framework**
Citation:	Wandersman A, Duffy J, Flaspohler P, et al. Bridging the gap between prevention research and practice: the interactive systems framework for dissemination and

	implementation. Am J Community Psychol 2008;41(3-4):171-81.
Website:	Not applicable
Name:	**IOWA Model of Evidence-based Practice**
Citation:	Titler MG, Kleiber C, Steelman VJ, Rakel BA, Budreau G, Everett LQ, Buckwalter KC, Tripp-Reimer T, Goode CJ. The Iowa model of evidence-based practice to promote quality care. Crit Care Nurs Clin North Am 2001;13:497-509.
Website:	Not applicable
Name:	**Kern's Medical Model for Curriculum Development**
Citation:	Thomas PA, Kern DE, Hughes MT, Chen BY (editors). Curriculum development for medical education: a six-step approach. 3rd ed. Baltimore: Johns Hopkins University Press; 2016.
Website:	Not applicable
Name:	**Knowledge-to-Action Framework**
Citation:	Graham ID, Logan J, Harrison MB, Straus SE, Tetroe J, Caswell W, Robinson N. Lost in knowledge translation: time for a map? J Contin Educ Health Prof 2006;26:13-24.
Website:	Not applicable
Name:	**Lavis' Framework for Knowledge Transfer**
Citation:	Lavis JN, Robertson D, Woodside JM, McLeod CB, Abelson J. How can research organizations more effectively transfer research knowledge to decision makers? Milbank Q 2003;81(2):221-2.
Website:	Not applicable
Name:	**Lewin's Change Theory**
Citation:	Lewin K. Frontiers in group dynamics: concept, method and reality in social science; social equilibria and social change. Hum Relat 1947;1:5-41.
Website:	Not applicable
Name:	**NHS Sustainability Model**
Citation:	Maher L, Gustafson D, Evans A. NHS Institute for Innovation and Improvement sustainability model and guide. Available from: https://improvement.nhs.uk/resources/Sustainability-model-and-guide/
Website:	Not applicable
Name:	**Normalization Process Theory**
Citation:	May CR, Mair F, Finch T, MacFarlane A, Dowrick C, Treweek S, Rapley T, Ballini L, Ong BN, Rogers A, Murray E, Elwyn G, Légaré F, Gunn J, Montori VM. Development of a theory of implementation and integration: Normalization Process Theory. Implement Sci 2009;4:29.
Website:	www.normalizationprocess.org
Name:	**Plan-Do-Study-Act (PDSA) Cycles**
Citation:	Langley GJ, Moen RD, Nolan KM, Nolan TW, Norman CL, Provost LP. The improvement guide: a practical approach to enhancing organizational performance. 2nd ed. San Francisco: Jossey-Bass; 2009.
Website:	Not applicable
Name:	**Practical Robust Implementation and Sustainability Model (PRISM)**
Citation:	Feldstein AC, Glasgow RE. A practical, robust implementation and sustainability model (PRISM) for integrating research findings into practice. J Comm J Qual Patient Saf 2008;34(4):228-43.

Website:	Not applicable
Name:	**Proctor's Implementation Outcome Framework**
Citation:	Proctor E, Silmere H, RaghaVan R, Hovmand P, Aarons G, Bunger A, Griffey R, Hensley M. Outcomes for implementation research: conceptual distinctions, measurement challenges, and research agenda. Adm Policy Ment Health 2011;38:65-76.
Website:	Not applicable
Name:	**Promoting Action on Research Implementation in Health Services (PARIHS)**
Citation:	Kitson A, Harvey G, McCormack B. Enabling the implementation of evidence based practice: a conceptual framework. Qual Health Care 1998;7(3):149-58.
Website:	Not applicable
Name:	**Quality Implementation Framework**
Citation:	Meyers DC, Durlak JA, Wandersman A. The quality implementation framework: a synthesis of critical steps in the implementation process. Am J Community Psychol 2012;50(3-4):462-80.
Website:	Not applicable
Name:	**Quality Enhancement Research Initiative (QUERI) Model**
Citation:	Demakis JG, McQueen L, Kizer KW, Feussner JR. Quality Enhancement Research Initiative (QUERI): a collaboration between research and clinical practice. Med Care 2000;38(6 Suppl 1):I17-25.
Website:	Not applicable
Name:	**Reach Effectiveness Adoption Implementation Maintenance (RE-AIM)**
Citation:	Glasgow RE, Vogt TM, Boles SM. Evaluating the public health impact of health promotion interventions: the RE-AIM framework. Am J Publ Health 1999;89(9):1322-7.
Website:	www.RE-AIM.org
Name:	**Replicating Effective Programs Model**
Citation:	Kilbourne AM, Neumann MS, Pincus HA, Bauer MS, Stall R. Implementing evidence-based interventions in health care: application of the replicating effective programs framework. Implement Sci 2007;2:42.
Website:	Not applicable
Name:	**Social Cognitive Theory**
Citation:	Bandura A. Social foundations of thought and action: a social-cognitive theory. Englewood Cliffs: Prentice-Hall; 1986.
Website:	Not applicable
Name:	**Star Model of Knowledge Transformation**
Citation:	Stevens KR. ACE Star Model of EBP: Knowledge Transformation. Academic Center for Evidence-based Practice. 2004 The University of Texas Health Science Center at San Antonio.
Website:	www.acestar.uthscsa.edu
Name:	**Theoretical Domains Framework**
Citation:	Michie S, Johnston M, Abraham C, Lawton R, Parker D, Walker A, on behalf of the Psychological Theory Group. Making psychological theory useful for implementing evidence based practice: a consensus approach. Qual Saf Health Care. 2005;14:26–33.
Website:	Not applicable

Name:	**Transtheoretical Model of Behaviour Change**
Citation:	Prochaska JO, DiClemente CC. Transtheoretical therapy: toward a more integrative model of change. Psychol Psychother 1982;19(3):276–88.
Website:	Not applicable
